# Marital Satisfaction: The Differential Impact of Social Support Dependent on Situation and Gender in Medical Staff in Iran

**DOI:** 10.5539/gjhs.v5n4p151

**Published:** 2013-05-12

**Authors:** Arian Rostami, Mehdi Ghazinour, Jörg Richter

**Affiliations:** 1 Department of Social Work, Umeå University, Umeå, Sweden; 2 Centre for Child and Adolescent Mental Health, Oslo, Norway

**Keywords:** marital satisfaction, social support, job satisfaction, medical staff, Iran

## Abstract

Stress is unavoidable in everyday life and it can effect on marital relationship. Social support especially from emotionally closed persons as a protective factor can help individuals to deal with stress and buffers the negative effects of life stress on marital satisfaction. In the present cross-sectional study we investigated the relationship between social and spousal support and marital satisfaction in medical staff in Iran. Data collection was performed in 653 medical staff using socio-demographic questions, the ENRICH Marital Satisfaction Inventory, and the Social Support Questionnaire. Women and men did not differ in total social support satisfaction and the total number of supporting people; but, women were more often support providers for their husbands than men were for their wives. Spouse support was a more important indicator of marital satisfaction for women than for men. Also results revealed that spouse support is more important than social support from other resources to explain marital satisfaction. Job satisfaction had an explanatory effect on marital satisfaction especially in men. Furthermore, the findings showed that social support could decrease the explanatory impact of job satisfaction on scales of marital satisfaction. Therefore, focusing on social support, especially spouse support could be an effective approach in family counseling or family education programs to improve marital satisfaction in medical staff.

## 1. Introduction

Acute and chronic stressful experiences significantly influence the development of close relationships and marital satisfaction (Neff & Karney, 2004, 2005; Story & Bradbury, 2004). The effect of stress on marital relationship can be in three ways; negative effect on couple's communication, decrease in time spend together, and increase in health problems. Stress protective factors can emerge from within individuals (e.g., coping, resilience), couples (e.g. marital communication) and outside family contexts (Patterson, 2002). Social support as a protective factor has effect in face of psychosocial stress (stress, social support and the buffering hypothesis). Particularly support from emotionally close persons can provide psychological resources needed to cope with stress (Cohen, 2004) and buffer negative effects of life stress on marital satisfaction (Chi et al., 2011). Social support refers to the function and quality of social relationships which can be as perceived support or actual received support (Sarason, Levine, Basham, & Sarason, 1983; Schwarzer & Leppin, 1991). Perceived social support has been defined as “an individual's perceptions of general support or specific supportive behaviors (available or enacted on) from people in their social network, which enhances their functioning or may buffer them from adverse outcomes” (Demaray & Malecki, 2002).

Social support includes providing instrumental support (actual help in time, money, and energy), informational support (information, suggestion and advice), appraisal support (evaluative feedback) and emotional support (empathy, trust, caring and love) (House, 1981). Women often have a greater number of close relationships and also a more extensive social network than men (Laireiter & Baumann, 1992; McFarlane, Neale, Norman, Roy, & Streiner, 1981). Additionally, women provide more emotional support to others; and they seek and receive more social support (Ashton & Fuehrer, 1993; Klauer & Winkeler, 2002; Reevy, 2007). Also, they benefit from the stress-buffering effects of social support more than men (Bellman, Forster, Still, & Cooper, 2003). Women emphasize intimacy and self-disclosure in their close relationships, and they are more empathetic, expressive, and disclosing than men.

Social support is one of the most important factors in marital relationships (Acitelli, 1996; Gottlieb, 1994). Studies found positive associations between satisfaction with spousal support and marital satisfaction (Acitelli, 1996; Julien & Markman, 1991; Pasch & Bradbury, 1998). Spouses expect partners to “be there” for help to solve problems, as well as providing consolation when problems lack solutions. Although both husbands and wives do turn to others in their social networks for various types of support, spouses remain an important source of support (Beach, Martin, Blum, & Roman, 1993; Walen & Lachman, 2000). Indeed, findings supported that social support from sources outside the marriage cannot compensate the lack of spousal support (Coyne & Delongis, 1986; Lieberman, 1982). Belle (1982) hypothesized that women provide more support than they receive in their marital relationship. Some studies also suggest that men and women differ in their perceptions of spouses’ support. Vinokur and Vinokur-Kaplan (1990) and Vanfossen (1981) found that husbands rate their spouses more supportive than did wives. In contrast, some other studies yielded results inconsistent with the support gap hypothesis. They revealed that men and women often report that they received similar types and amounts of support from partners (DeLongis, Capreol, Holtzman, O’Brien, & Campbell, 2004; Neff & Karney, 2005).

Medical staff faces many stressors in their job. Patients’ pain and suffering, work time pressure, heavy workload, inadequate salary, and inequality at work are perceived as major sources of stress by hospital employees (Adeb-Saeedi, 2002; Cory, 2007; Mosadeghrad, Ferlie, & Rosenberg, 2011; Roberts & Levenson, 2001). Studies indicated that job stress can affect negatively on individuals’ quality of life, but also on their marital relationship (Ardekani, Kakooei, Ayattollahi, Choobineh, & Seraji, 2008; Bodenmann, 2000; Hamaideh, 2012; Mauno & Kinnunen, 1999; Serafini, 2010; Su, Weng, Tsang, & Wu, 2009; Wu, Li, Wang, Yang, & Qiu, 2011). Job dissatisfaction or being employed in a high demanding job as an everyday stress can negatively influence individual's wellbeing, quality of relationships and marital satisfaction (R. Hill, 1958; Hochschild, 1997; D. Hughes, Galinsky, & Morris, 1992; D. L. Hughes & Galinsky, 1994; Rogers & May, 2003). Job, family and individual characteristics stressors (for example; job pressure, job hours, and household duty) as A (stressors) in the ABCX model (R. Hill, 1958; McCubbin & Patterson, 1983) in family stress theory and work, family, individual characteristics resources and support as B (recourses and support) results the perception of work-family conflict and facilitation as corresponding to C. Theoretically, interactions of these three lead to outcome (X). In this model job satisfaction is identified as work outcomes; family satisfaction and marital satisfaction as family outcomes; and life satisfaction and individual stress as individual outcome (Hill, 2005). Then, based on this model social support as a moderator can improve physical and psychological health, facilitate couples’ marital satisfaction and job satisfaction in medical staff that are under the pressure of job stress (Aycan & Eskin, 2005; Button, 2008).

In the present study, we investigated the relationship between social and spousal support and marital satisfaction in medical staff in Iran. The objective of our study was to analyse: 1) social and spousal support in different situations among medical staff; 2) gender differences in social and spousal support; 3) associations between social and spousal support and marital satisfaction by gender; and (d) The moderator effect of socio-demographic variables, job satisfaction and social and spousal support dependent on the domains of marital satisfaction.

## 2. Material and Methods

### 2.1 Sample and Procedure

This was a cross-sectional study on social and spouse support and marital satisfaction in 653 medical staff in hospitals that are affiliated to Tehran Medical University. The sample comprised of married (for at least one year) medical staff, who lived together with their spouse; who did not had any addiction problems or severe physical and psychological disorders which could affect their life (see Table 1 for characteristics of the sample).

**Table 1:** Socio-demographic variables by gender

	Females	Males	t	Df	p
N	477	175			
Years married	9.29±7.19	10.74±7.52	-2.20	298	.028
Age	35.36±7.14	39.22±7.70	-5.79	291	< .001
Number of children	0.97±0.97	1.31±0.95	-4.09	650	< .001
Spouse’s age	38.13±8.25	34.68±6.95	5.33	365	< .001
Job-Satisfaction	2.91±0.92	3.16±1.19	-2.54	255	.012
Number of Children	0.99±0.93	1.26±1.07			
Education of the subject (N)					
HND	102 (21.4 %)	57 (32-6 %)	χ^2^ 26.44		< 0.001
Bachelor	317 (66.5 %)	78 (44.5 %)			
Master or higher	58 (12.2 %)	40 (22.9 %)			
Education of the spouse					
HND	194 (40.7 %)	62 (35.4 %)	χ^2^ 2.87		0.238
Bachelor	199 (41.7 %)	86 (49.1%)			
Master or higher	84 (17.6 %)	27 (15.4 %)			
Employment of the spouse					
Employed	447 (93.7 %)	101 (57.7 %)	χ^2^		
Not employed	30 (6.3 %)	74 (42.3 %)	123.74		< 0.001

HND: Higher National Diploma

The study was approved by the scientific and Ethics committee of Tehran Medical University, Iran. After receiving the permission to perform the investigation from the Tehran Medical University and hospital managers, the aims of study were explained to the staff of every hospital ward by the principle researcher. Participants were asked to complete a set of questionnaires. Participation in the study was voluntary; and the participants could withdraw from the investigation at any time of the investigation.

### 2.2 Instruments

Data was collected by a set of questionnaire which was split into three parts; a socio-demographic form, the ENRICH marital satisfaction inventory, and the Social Support Questionnaire (SSQ).

The **Socio-demographic questionnaire** was designed by the principal researcher to collect socio-demographic information such as participant's age, gender, education, length of marriage, number of children, spouse's age, education, job satisfaction (single, general question to be answered on a 5-point scale between 1 very dissatisfied and 5 very satisfied) and some other socio-demographic variables.

The **ENRICH Marital Satisfaction inventory** (Evaluating & Nurturing Relationship Issues, Communication, and Happiness) is a multidimensional self-report measurement of marital satisfaction that was developed by Olson and colleagues (Olson, Fournier, & Druckman, 1983). It includes 125 items to be answered on a 5-point Likert scale ranging from very dissatisfied to very satisfied) grouped into 14 domains; Idealistic Distortion, Marital Satisfaction, Personality Issues, Communication, Conflict Resolution, Financial Management, Leisure Activities, Sexual Relationship, Children and Parenting, Family and Friends, Equalitarian Roles, Religious Orientation, Marital Cohesion and Marital Change (Fowers & Olson, 1989). The short form of this questionnaire that was standardized by Soleimanian (1994) was applied for assessing marital satisfaction in the present study. This version consists of 47 items in 9 scales: personality issues, marital communication, conflict resolution, financial management, pleasure activities, sexual activities, marriage and children, family and friends, and religious orientation. Construct validity by comparison with a Family Satisfaction Scale showed an acceptable level of shared variance (0.41-0.60) between the scales. Internal consistency for the measure was calculated as Cronbach's alpha of 0.95 for men and women. Furthermore test-retest reliability was measured with a reliability coefficient of 0.92 (Rasooli, 2001).

The **Social Support Questionnaire** (**SSQ)** (Sarason et al., 1983) is a self-report questionnaire consisting of 27 scenarios. In relation to each scenario, respondents are asked: (a) to report any persons who would be accessible for support in that circumstance and (b) how satisfied they would be with the available support for that specific situation on a 6-point Likert scale ranging from 1 (very dissatisfied) to 6 (very satisfied). Based on the responses to the two questions two scores are originally calculated: the average number of available supportive individuals (Social Support Questionnaire Number—SSQN) and the average satisfaction with the available SS (Social Support Questionnaire satisfaction—SSQS). The validity and reliability of the Persian version of the SSQ was satisfactory (SSQN α = 0.95 and SSQS α = 0.96) (Nasseh, Ghazinour, Joghataei, Nojomi, & Richter, 2011). We additionally created a third scale representing the frequency of reporting the spouse as a supporting person. Furthermore, the scenarios of the SSQ were grouped for this study according to the type of situation by expert ratings (six clinical psychologists and social workers). Six domains were derived: Need for self-disclosure (scenarios 1, 6, 11, 14, 21); Need for support in loss situations (scenarios 2, 4, 10, 18); Need for belonging (scenarios 3, 20, 24); Need for instrumental support (scenarios 5, 8, 9, 13, 26); Need to be praised (scenarios 7, 12, 19, 22); and Need for emotional support (scenarios 15, 16, 17, 23, 25, 27). The average number of supporting persons, the average satisfaction with the support and the average frequency of reporting the spouse as a supporting person were computed for each of the domains.

### 2.3 Statistical Analysis

Descriptives of the assessed variables are reported in terms of mean scores and standard deviations or percentages dependent on the scale level by gender. T-test was used for testing for group differences for continuous variables and χ^2^-test for categorical variables. Pearson correlation coefficients are provided to indicate associations between continuous variables. Hierarchical multiple regression analysis was used to test for the predictive value of socio-demographic variables in a first block (method: enter) and social support related variables in a second block (method: stepwise) with marital satisfaction domains as dependent variables. All the calculations were run by gender. The analysis was performed by SPSS 19 for PC and Mac.

## 3. Results

### 3.1 Marital Satisfaction

Male medical staff members reported an overall significant higher marital satisfaction than the females mainly based on that they were more satisfied in the domains of personality issues, conflict resolution, sexual activities, family and friends, marital communication and Marriage and Children (Table 2).

**Table 2:** Marital satisfaction domains by gender

	Females	Males	t	df	p
Personality Issues	16.75±4.45	18.18±4.43	-3.65	650	< .001
Marital Communication	17.16±4.42	17.98±4.32	-2.10	650	.036
Conflict Resolution	17.07±4.01	18.15±3.41	-3.42	361	.001
Financial Management	18.14±4.17	18.64±3.25	-1.64	395	.102
Pleasure Activities	17.67±3.53	17.79±3.61	-0.39	650	.698
Sexual Activities	17.61±3.69	18.71±3.30	-3.44	650	.001
Marriage & Children	17.21±3.82	18.10±4.43	-2.08	426	.038
Family & Friends	17.02±3.36	17.77±3.44	-2.50	650	.013
Religious Orientation	18.61±3.98	18.99±3.76	-1.07	650	.285
Marital Satisfaction Total	158.54±27.37	166.71±26.82	-3.40	650	.001

### 3.2 Social Support

Neither the average total number of supporting persons, nor the average total satisfaction with perceived social support differed between the genders. However, men reported significantly more often their wives as supporting persons than women named their husbands as supporting.

Furthermore, men reported their wives as supporting persons significantly more often than women in scenarios related to need for belongingness, need for emotional support, need for self-disclosure, need to be praised, and need for instrumental support, and they evaluated their satisfaction with social support related to needs for belongingness substantially higher than the women. Women evaluated their satisfaction with social support in loss situations higher than the men (Table 3).

**Table 3:** Social support domains by gender

	Females	Males	t	df	p
Total Social Support Number	2.01±1.11	1.94±1.09	0.69	650	.493
Total Social Support Satisfaction	4.81±1.07	4.66±1.30	1.50	650	.133
Spouse Support Total	15.56±8.34	17.85±6.88	-3.45	352	.001
Need for Self-Disclosure Number	2.03±1.11	2.17±1.23	-1.34	639	.181
Self-Disclosure Satisfaction	4.87±0.96	4.94±0.95	-0.86	639	.389
Self-Disclosure Spouse	0.58±0.32	0.67±0.29	-3.45	639	.001
Support in Loss Situations Number	1.54±1.33	1.46±1.30	0.64	639	.521
Loss Situations Satisfaction	4.46±1.26	4.04±1.24	3.67	639	< .001
Loss Situations Spouse	0.35±0.29	0.31±0.29	1.45	639	.148
Need for Belongingness Number	2.82±1.51	2.92±1.66	-0.67	271	.501
Belongingness Satisfaction	5.10±0.90	5.27±0.83	-2.19	313	.029
Belongingness Spouse	0.70±0.35	0.82±0.32	-4.29	324	< .001
Need for Instrumental Support Number	2.06±1.17	1.98±1.10	0.74	639	.458
Instrumental Support Satisfaction	4.99±0.95	4.90±1.06	1.06	639	.291
Instrumental Support Spouse	0.60±0.37	0.67±0.34	-2.12	322	.035
Need to be Praised Number	2.02±1.24	1.86±1.07	1.49	639	.137
Be Praised Satisfaction	4.87±1.06	4.82±1.21	0.52	264	.603
Be Praised Spouse	0.56±0.36	0.66±0.33	-2.98	639	.003
Need for Emotional Support Number	1.83±1.20	1.77±1.08	0.55	639	.582
Emotional Support Satisfaction	3.89±0.90	3.95±0.86	-.879	639	.385
Emotional Support Spouse	0.50±0.31	0.60±0.25	-4.22	363	< .001

### 3.3 Associations between Marital Satisfaction Domains and Social Support Domains

The correlation between the average number of supporting persons and reporting the partner as supporting were of moderate to large effect size among the females staff with an average coefficient r = 0.47 (ranging from needs for instrumental support r = 0.42 to r = 0.52 for scenarios related to needs for emotional support) and of small to moderate effect size among males r = 0.32 (ranging from needs for emotional support r = 0.16 to r = 0.35 for scenarios related to needs to be praised) with a significant gender difference (p = 0.045). There was no difference between the average correlation between satisfaction with social support and naming the partner as supporting person which were of moderate to high effect size (women: r = 0.52 - ranging from needs in loss situations r = 0.49 to r = 0.59 for scenarios related to needs for emotional support; men: r = 0.49 - ranging from needs in loss situations r = 0.37 to r = 0.59 for scenarios related to needs to be praised).

### 3.4 Prediction of Marital Satisfaction based on Social Support

In hierarchical regression analyses by gender (Tables 4a, b) with the various marital satisfaction domain scores as dependent variables and socio-demographic variables of impact (age, spouse's age, years married, subject's and partner's education, job and job satisfaction) included in the first block (method: enter) and the average number of supporting persons, the average satisfaction with social support, and the average frequency of reporting the partner as a source of support related to the six scenario-groups derived from the SSQ in the second block (method: stepwise) as independent variables a more differentiated pattern appeared:

**Table 4a:** Hierarchical multiple regressions with social support domain scores as independent and marital satisfaction domains as dependent variables controlling for socio-demographic variables (Block 1) (1^st^ block: method enter; 2^nd^ block: method stepwise) in females

Independent	Adjusted r^2^	F	p	Variables with significant standardized Beta (β, t, p)
Personality Issues				
Block 1	0.10		< 0.001	
Block 2	0.37	31.85	< 0.001	Instrumental spouse (0.36; 6.10; < 0.001); belongingness spouse (0.22; 3.77; <0.001)

Marital Communication				
Block 1	0.14		< 0.001	
Block 2	0.36	30.30	< 0.001	Instrumental spouse (0.28; 4.76; <0.001); belongingness spouse (0.24; 4.03; <0.001);

Conflict Resolution				
Block 1	0.06		< 0.001	
Block 2	0.34	27.84	< 0.001	Belongingness spouse (0.39; 6.79; < 0.001); praise spouse (0.19; 3.24; 0.001)

Financial Management				
Block 1	0.16		< 0.001	
Block 2	0.36	30.30	< 0.001	Instrumental spouse (0.34; 5.76; < 0.001); belongingness spouse (0.15; 2.62; 0.009)

Pleasure Activities				
Block 1	0.06		< 0.001	
Block 2	0.23	13.65	< 0.001	Emotional spouse (0.35; 6.59; <0.001); instrumental no. (-0.25; -3.57; <0.001); praise no. (0.21; 2.95; 0.003); praise satisfaction (0.12; 2.26; 0.024)

Sexual Activities				
Block 1	0.07		< 0.001	
Block 2	0.22	15.18	< 0.001	Belongingness spouse (0.26; 4.208; <0.001); praise spouse (0.16; 2.541; 0.012);

Marriage and Children				
Block 1	0.03		0.039	
Block 2	0.13	6.02	< 0.001	Praise spouse (0.18; 2.03; 0.043); instrumental spouse (-0.19; 2.09; 0.037)

Family & Friends				
Block 1	0.06		< 0.001	
Block 2	0.14	8.05	< 0.001	Praise no. (0.31; 4.13; <0.001); instrumental no. (-.28; -3.72; <0.001); instrumental spouse (0.18; 3.38; 0.001); emotional satisfaction (0.10; 1.95; 0.052)

Religious Orientation				
Block 1	0.06		< 0.001	
Block 2	0.23	13.61	< 0.001	Instrumental spouse (0.36; 8.16; <0.001); self-disclosure no. (0.13; 2.12; 0.035); loss no. (-0.26; -3.28; 0.001); loss spouse (0.24; 3.59; <0.001)

Job Satisfaction				
	0.14	9.16	< 0.001	Age (β = -.41; t = -3.80; p > .001); spouse age (β = .49; t = 4.69; p > .001); spouse education (β = .14; t = 2.78; p = .006); spouse job (β = -.13; t = -2.87; p = .004); praise satisfaction (0.21; 4.11; <0.001); self-disclosure no. (-0.09; -2.06; 0.040), instrumental spouse (-0.10; -2.02; 0.044)

**Table 4b:** Hierarchical multiple regressions with social support domain scores as independent and marital satisfaction domains as dependent variables controlling for socio-demographic variables (Block 1) (1^st^ block: method enter; 2^nd^ block: method stepwise) in males

Independent	Adjusted r^2^	F	p	Variables with significant standardized Beta (β, t, p)
Personality Issues				
Block 1	0.04		0.058	
Block 2	0.31	6.90	< 0.001	Belongingness spouse (0.35; 3.60; < 0.001); emotional spouse (0.21; 2.15; 0.033); praise no. (0.28; 2.27; 0.024); loss satisfaction (0.17; 2.37; 0.019); instrumental no. (-0.36; -3.04; 0.003)

Marital Communication				
Block 1	0.06		0.027	
Block 2	0.42	10.76	< 0.001	Belongingness spouse (0.54; 6.544; < 0.001); emotional satisfaction (0.33; 4.68; <0.001); loss satisfaction (0.18; 2.75; 0.007); emotional no. (-0.20; -2.78; 0.006); instrumental spouse (-0.17; -1.98; 0.049)

Conflict Resolution				
Block 1	0.02		0.171	
Block 2	0.31	7.49	< 0.001	Belongingness spouse (0.53; 7.09; < 0.001); praise no. (0.28; 2.32; 0.022); instrumental no. (-0.42; -3.59; <0.001); self-disclosure no. (0.27; 3.46; 0.001)

Financial Management				
Block 1	0. 08		0.006	
Block 2	0.34	7.42	< 0.001	Belongingness spouse (0.32; 3.31; 0.001); praise satisfaction (0.22; 2.54; 0.012); self-disclosure spouse (-0.19; -2.58; 0.011); self-disclosure satisfaction (0.22; 2.91; 0.004); emotional spouse (0.32; 2.97; 0.003); praise spouse (-0.32; -3.02; 0.003)

Pleasure Activities				
Block 1	0.12		< 0.001	
Block 2	0.32	8.55	< 0.001	Belongingness spouse (0.33; 4.337; <0.001); emotional satisfaction (0.30; 4.14; <0.001); instrumental no. (-0.15; -2.03; 0.044)

Sexual Activities				
Block 1	0.12		< 0.001	
Block 2	0.43	12.00	< 0.001	Belongingness no. (-0.44; -5.99; <0.001); praise spouse (0.28; 3.45; 0.001); self-disclosure spouse (-0.19; -2.92; 0.004); belongingness spouse (0.32; 3.58; <0.001)

Marriage & Children				
Block 1	0.20		< 0.001	
Block 2	0.57	14.10	< 0.001	Belongingness satisfaction (0.57; 5.180; <0.001); belongingness spouse (0.33; 3.68; <0.001); instrumental satisfaction (-0.30; -2.54; 0.013); emotional no. (-0.28; -3.58; 0.001);

Family & Friends				
Block 1	0.06		0.017	
Block 2	0.41	10.06	< 0.001	Belongingness spouse (0.20; 2.07; 0.041); instrumental no. (-0.45; -6.06; <0.001); belongingness satisfaction (0.28; 3.47; 0.001); emotional spouse (0.25; 2.72; 0.007); loss satisfaction (0.16; 2.43; 0.016)

Religious Orientation				
Block 1	0.11		0.001	
Block 2	0.39	10.44	< 0.001	Praise satisfaction (0.21; 2.80; 0.006); belongingness spouse (0.40; 5.59; <0.001); loss satisfaction (0.28; 4.16; <0.001); instrumental no. (-0.24; -3.27; 0.001)

Job satisfaction				
	0.22	6.54	< 0.001	Age (β = -.39; t = -2.12; p = .036); education (β = .54; t = 5.085; p < .001); belongingness spouse (0.24; 3.22; 0.002); emotional no. (0.24; 3.02; 0.001)

a) the variance of the socio-demographic variables from block 1 explained between 3 % of the variance in Marriage & Children and 16 % in Financial Management among the female medical staff; and between 2 % in Conflict Resolution and 20 % in Marriage & Children in the male staff;
b) the variance of the variables from block 2 (varying number and varying variables related to social support) additionally explained between 8 % of the variance in Family and Friends and 28 % in Conflict Resolution among the females and between 20 % in Pleasure Activities and 37 % in Marriage & Children among the males;
c) the total amount of variance explained by both blocks together varied between 13 % in Marriage & Children and 37 % in Personality issues for females and between 31 % in Personality Issues and in Marital Communication and 57 % in Marriage & Children for males;
d) for the males, only 41.5 % of the significantly contributing variables remaining in the regression equation were related to spouse support, whereas these were 66.7 % for the women; and
e) social support from the husband in situations characterized by need for instrumental support and by need for self-disclosure was most often of predictive impact related to marital satisfaction among the females, whereas support by their wife in situations of needs for belongingness was most often of predictive impact among the males.

### 3.5 Associations between Job Satisfaction, Marital Satisfaction and Social Support

When separating job satisfaction from the socio-demographic variables as a second block within these calculations, it additionally explained between 0 % (women: in Pleasure Activities and Sexual Activities; men: Personality Issues and Conflict Resolution) and 3 % of variance among the women in Family & Friends and 5 % in Marriage & Children among the men when controlling for the impact of the various social-demographic variables in block 1 (findings not presented in a table). After inclusion of the various social support variables into the regression as a third block, job satisfaction kept its variance explaining power for marital satisfaction scales among the women for Personality issues (β = .17; t = 4.32; p > .001), Marital Communication (β = .09; t = 2.26; p = .024), Financial Management (β = .09; t = 2.36; p = .019), Family & Friends (β = .17; t = 3.67; p > .001), and Religious Orientation (β = .12; t = 2.67; p = .008) as well as for Marriage & Children (β = .21; t = 2.70; p = .0008) and Family & Friends (0.17; 2.48; 0.014) for men. However, its explanatory impact lost its significance when including the social support variables into the equation for scales Financial Management, Pleasure Activities, Sexual Activities, and Religious Orientations among men.

Fourteen percent of the variance in job satisfaction could be explained by the variation in socio-demographic variables and the various social support variables among the women and 22 % among the men (see Table 4a, b).

### 3.6 Prediction of Marital Satisfaction by Total Social Support (findings not presented in a table)

When replacing the differentiated social support scores based on the situations of need for support by the three total social support scores (number of supporting persons, satisfaction with support, spouse support) within the regression equation, the variation in the variables in both blocks together explained between 11 % (Family and Friends) and 36 % (Personality Issues) for the women; and, between 18 % (Family and Friends) and 26 % (Marriage & Children and Religious Orientation) in the men. Spouse support was the only significant variable of the three social support scores remaining as significantly explaining variance in the ENRICH scores among the women with percentages between 5 % of Family & Friends and 26 % of Personality Issues. In the men's data, spouse support alone explained between 6 % of variance in Marriage & Children and 17 % in Personality Issues and Conflict Resolution.

## 4. Discussion

The general aim of our investigation was to assess the relationship between social and spousal support and marital satisfaction in Iranian medical staff. Our findings confirmed previous results that men usually report higher marital satisfaction than do women (Jose & Alfons, 2007; Ng, Loy, Gudmunson, & Cheong, 2009) even though they did not differ on satisfaction with social support. Today, Iranian women are still the main responsible for the majority of household duties even if they have a job outside the family (Rafatjah, 2011). Therefore, women usually perceive more stress because of additional duties related to childcare and other household tasks (for more also see Rostami, Ghazinour, Nygren, & Richter, 2013).

Results showed that although the total social support satisfaction and the total number of supporting people were not different between men and women; women were more often perceived as support providers for their husbands than men were for their wives. This finding corresponds to the “support-gap hypothesis” (Belle, 1982) which notions that women receive less support from their spouses than men receive from their spouses. This difference can also be explained by social and cultural expectations from women to be more supportive and providers of nurturance and support (Barbee et al., 1993; Schwarzer & Gutierrez-Dona, 2005; Wood, 1994) and for men by the masculine role according to which men are assumed to behave more independently and self-reliant (Deaux & Marianne, 1998). This social expectation guides women to seek and provide support more often and better than men. Based on an investigation among Iranian couples, Izadi and colleagues (2010) revealed that women expect their husbands to express their love and support both verbally and behaviorally. Iranian women expect their husband to be romantic and emotional; to remember their special occasions such as birthday or wedding anniversary; to express their love directly in words; and also to collaborate in household chores and child rearing. However, Iranian men prefer to express their feelings in rather indirect behavioral ways such as attempts of providing the family with a better economical or practical situation. This obvious discrepancy related to expectancies may cause Iranian women experiencing less spouse support than men. Because of the ways men support their wives do not match the women's expectancies, the women often feel that their husbands don’t care about them as much as they wish and perceive inadequate and dissatisfactory support from their husbands. Another interpretation from an empowerment perspective could be that male respondents in our sample are more passive supportive whereas female respondents are more active in giving support.

Spouse support was a more important indicator of marital satisfaction for women than for men. Several studies have shown that the link between husband's support and marital satisfaction in women is stronger than the link between wives’ support and marital satisfaction in men (Acitelli & Antonucci, 1994; Julien & Markman, 1991). Also other studies showed that some husbands’ support skills (e.g., relationship awareness, expressiveness, intimacy, maturity) are more predictive of relationship and life satisfaction in women than in men (Acitelli & Antonucci, 1994; Lamke, 1989; Murstein & Williams, 1985; White, Speisman, Jackson, Bartis, & Costos, 1986). Indeed, findings showed spouse support is more important than social support from other resources to explain marital satisfaction. Support from partner is uniquely beneficial. In other words, inadequate spousal support isn’t compensated by support from other sources (Coyne & Delongis, 1986; DeLongis et al., 2004). When couples perceive their spouse as the main resource of support, the family cohesion and the emotional bonds between couples become stronger and this can lead to the higher marital satisfaction.

Furthermore, support in situations of need for instrumental support or self-disclosure support from husbands among women and in situations of need for belongingness support from wives among men were most predictive related to marital satisfaction. Women tend to be more self-disclosing than man; and they also tend more positively to assess their marital satisfaction by their self-disclosure about personal facts, feeling and communication (Peplau & Gordon, 1985). Thus, spouse support in self-disclosure can be a more important factor related to women's marital satisfaction than men's. Monadi's study (2004) among Iranian couples in Tehran indicated that disclosing had an important effect on marital satisfaction especially in women. Women were more satisfied when their husbands listened and cared about their disclosing. They like to talk about the problems and stressful events of life and expect their husbands to care about that even if they can’t solve the problem.

Also, as Mickelson and colleagues (2006) reported, instrumental support in addition to emotional support predicted marital satisfaction in women with an egalitarian gender role belief. Since the women of the current study were an educated and socially active group, it seems that the spouse's instrumental support, for example, helping in household task and child rearing, is highly expected. Although in many of todays Iranian families women work outside, men are still the main responsible person for providing with the expenses of the family. Izadi and colleagues (2010) results showed that even educated and employed working Iranian women expected their husbands to provide the family expenses to cover their and their children's needs. Furthermore regarding house chores and child rearing, all women expected cooperation from their husbands. Since the women are still supposed to be responsible for household and children duty in Iranian families, the expectation of cooperation in these subjects could be a clash of expectations between couples in modern Iranian families. As our findings showed instrumental support is predicted as effective in many domains of marital satisfaction for example “financial management”, “marriage and children”, “communication”, “conflict resolution” and “personality issues”. It seems that women expect men to support them when they need consultation about their problems, help for family and children duties and even support as financial source of family.

Furthermore, support in situations of need for belongingness from wives among men was related to all domains of marital satisfaction. It means knowing that he was “an important part of his wife's life” and his wife “cared about him regardless of what is happening to him” and “loved him deeply”, could improve marital satisfaction in all aspects especially in “marriage and children” domain. Being convinced from the unconditional love of their wives and having the feeling that the wife has a deep sense of belongingness are important factors in marital satisfaction in men in this sample. Based on religion and cultural principals, women are still taught to be obedient, patient and supporting to her husband in Iran. Being the breadwinner of the family gives men higher authority in the family. About 65% of the women in our study were educated, employed (60 %), and economically independent. In this type of family with decreased men's economical authority and women's financial dependency, the emotional belonging of wives means more for men. In another words, the men feel more power and satisfaction when they feel that they have their wives’ love, concern and care.

The higher explanatory effect of job satisfaction in men could also be explained by the importance of a job for men compare to women. Men are supposed to be the main breadwinner in an Iranian family; and having a job is more important in men's life compared to women's taking them culturally and religiously determined role of men in Iranian families into account. The work domain is a greater source of problems for men than the family domain which is the greater source of conflicts for women. Therefore, the negative effect of job conflicts on the family domain in men is higher than among women (Karimi, 2009; Lambert, 1990; Posig & Kickul, 2004). Furthermore, the findings showed that social support could decrease the explanatory impact of job satisfaction on many marital satisfaction scales. The findings based on the family stress theory, as described by Hill (2005), confirm the importance of the moderating role of social support in effect of job satisfaction on marital satisfaction. Furthermore, Karimi and Nouri (2009) found that the family conflict because of a job has a negative relationship with perceived social support in a study in Iranian men.

Additionally, the explanatory impact of socio-demographic variables and social support on the variance of job satisfaction also confirmed the findings of other studies in Iran and western countries (Harris, Winskowski, & Engdahl, 2007; Veissi, Atefvahid, & Rezaee, 2000) and revealed that social support protects people from potentially negative influences of stressful events like the stress-buffer theory posits.

According to the results, cultural gender expectations, family stress (such as conflicts between couples, household and childrearing responsibilities), socio-demographic characteristics and job stress related to work as medical staff as stressors on one the hand and social and spousal support and socio-demographic characteristics as resources and support on the other hand determine perceived family stress and job stress by couples which consequently determined the level of perceived marital satisfaction and job satisfaction.

The present study had several limitations. Marital satisfaction and social support were assessed by only one partner of each couple, which made impossible comparisons within couples. Investigating dyadic marital satisfaction and social support would provide more detailed information especially about spouses support and its possible causality. The cross-sectional study design restricted the interpretation of the relationships between marital satisfaction and other factors. However, despite the limitations mentioned above, we believe that our findings should be considered by family counselors, psychologists and social workers and health policy makers especially at workplaces since there is a lack of knowledge about social support and marital satisfaction in Iran.
